# Cardioprotective Effect of Electroacupuncture Pretreatment on Myocardial Ischemia/Reperfusion Injury via Antiapoptotic Signaling

**DOI:** 10.1155/2016/4609784

**Published:** 2016-05-25

**Authors:** Sheng-feng Lu, Yan Huang, Ning Wang, Wei-xing Shen, Shu-ping Fu, Qian Li, Mei-ling Yu, Wan-xin Liu, Xia Chen, Xin-yue Jing, Bing-mei Zhu

**Affiliations:** ^1^Key Laboratory of Acupuncture and Medicine Research of Ministry of Education, Nanjing University of Chinese Medicine, Nanjing 210023, China; ^2^Key Laboratory of Acupuncture and Immunological Effects, Shanghai University of Traditional Chinese Medicine, Shanghai 200030, China; ^3^School of Veterinary Medicine, University of Pennsylvania, 3800 Spruce Street, Philadelphia, PA 19104, USA; ^4^Jiangxi University of Traditional Chinese Medicine, Nanchang 330004, China

## Abstract

*Objectives*. Our previous study has used RNA-seq technology to show that apoptotic molecules were involved in the myocardial protection of electroacupuncture pretreatment (EAP) on the ischemia/reperfusion (I/R) animal model. Therefore, this study was designed to investigate how EAP protects myocardium against myocardial I/R injury through antiapoptotic mechanism.* Methods*. By using rats with myocardial I/R, we ligated the left anterior descending artery (LAD) for 30 minutes followed by 4 hr of reperfusion after EAP at the Neiguan (PC6) acupoint for 12 days; we employed arrhythmia scores, serum myocardial enzymes, and cardiac troponin T (cTnT) to evaluate the cardioprotective effect. Heart tissues were harvested for western blot analyses for the expressions of pro- and antiapoptotic signaling molecules.* Results*. Our preliminary findings showed that EAP increased the survival of the animals along with declined arrhythmia scores and decreased CK, LDH, CK-Mb, and cTnT levels. Further analyses with the heart tissues detected reduced myocardial fiber damage, decreased number of apoptotic cells and the protein expressions of Cyt c and cleaved caspase 3, and the elevated level of Endo G and AIF after EAP intervention. At the same time, the protein expressions of antiapoptotic molecules, including Xiap, BclxL, and Bcl2, were obviously increased.* Conclusions*. The present study suggested that EAP protected the myocardium from I/R injury at least partially through the activation of endogenous antiapoptotic signaling.

## 1. Introduction

Acute myocardial ischemia (AMI) is the most common cause of mortality and morbidity in the developed countries and rapidly becoming a common malady in the developing countries. Early and fast restoration of blood flow is the most ideal approach to prevent further tissue injury [1]. In fact, thrombolytic therapy via drug administration or primary percutaneous coronary intervention (PCI) is the most effective strategy to reduce the size of myocardial infarct and improve clinical outcome. Unfortunately, however, prompt reperfusion can also induce myocardial injury, and the phenomenon is termed myocardial ischemia/reperfusion injury (MIRI) [2].

The damage from reperfusion is triggered by the increased production of oxygen-free radicals at the time of reperfusion and the impaired antioxidant ability of the heart, leading to cell apoptosis and increased infarct size [3, 4]; this accounts for up to 50% of the final size of a myocardial infarct [5]. To reduce MIRI, various methods and drugs have been used in experimental and clinical studies [6], such as remote ischemic preconditioning [7], exenatide [8], and atorvastatin pretreatment [9]. However, effective therapies to prevent reperfusion injury have proven elusive. Despite an improved understanding of the pathophysiology of this process and encouraging preclinical trials of multiple agents, most of the clinical trials to reduce MIRI have yielded disappointing results [10, 11]. Adjunctive therapies and new treatments to limit reperfusion injury remain an active area of investigation.

Acupuncture has been practiced in China for over two thousand years as an effective approach to improve the symptoms of angina and palpitation by promoting physiologically endogenous protection system [12–15]. According to Chinese medicine theory, Neiguan acupoint (PC6) on the pericardium meridian is considered as the main acupoint for improving heart function and energy metabolism, promoting ischemia tolerance, eliminating free radical, and protecting against cell death [16–24]. Growing evidence, including our previous study, experimentally shows that EAP could alleviate I/R injury of brain and heart tissues [15, 25–27]. Simultaneously, EAP also plays protective roles against cardiac I/R injury in adult patients undergoing heart valve replacement surgery by reducing the level of serum cardiac troponin I, the inotrope score, and by shortening time spent in the intensive care unit [22], and also by reducing PCI-related myocardial injury [28]. However, detailed mechanisms have not been fully elucidated.

The importance of apoptosis in cell death following reperfusion has been demonstrated in in vivo rodent models, which also allows for the evaluation of pharmacological, growth factor-mediated, and genetic interventions [2, 6]. Our previous study has used RNA-seq technology to show that apoptotic molecules were involved in the myocardial protection of EAP at PC6 on the I/R animal model [27], suggesting that cardioprotective effect of EAP may be closely related to antiapoptotic signaling. Therefore, the aims of our present study are to determine, through rat I/R models, whether EAP protects myocardium, along with its antiapoptotic mechanism. The results show that EAP could regulate both pro- and antiapoptotic molecules, suggesting that EAP might be an appropriate alternative treatment for myocardial ischemia patients who will be undergoing a thrombolytic therapy or primary percutaneous coronary intervention.

## 2. Methods

### 2.1. Antibodies and Reagents

Antibodies for Cyt c, Smac/Diablo, HtrA2/Omi, Endo G, AIF, caspase 3, cleaved caspase 3, Xiap, BclxL, and Bcl2 were obtained from cell signaling. Antibodies for GAPDH were purchased from Abcam (Cambridge, UK). For assessing apoptosis, the in situ cell death detection kit, POD (TUNEL) was obtained from Roche (Lewes, UK).

### 2.2. Animals and Grouping

**Figure 1:**
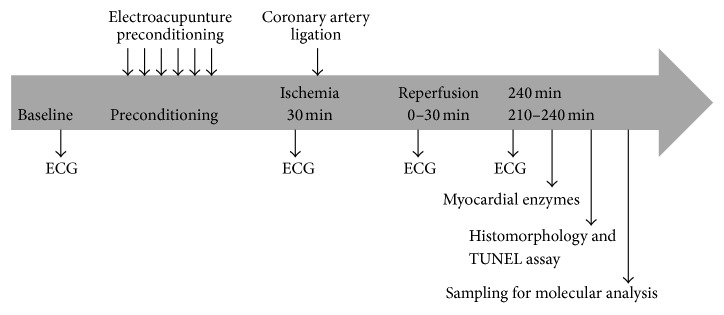
The timeline of the experimental process. Six preconditioning groups of rats were used (*n* = 15/each group). ECG indicates electrocardiogram.

Male adult Sprague-Dawley (SD) rats (280 ± 20 g), supplied by the Vital River Laboratory Animal Technology Co. Ltd. (Beijing, China), were randomly divided into six groups after adaptive feeding for a week: sham operation control group (SO), EAP at PC6 + SO (ES), EAP at nonacupoint + SO (NS), myocardial ischemia/reperfusion model group (I/R), EAP at PC6 + I/R (EA), and EAP at nonacupoint + I/R (NA). A timeline of the study is shown in Figure 1. The study was approved by the Institute for Animal Care and Use Committee at Nanjing University of Chinese Medicine, and all experimental procedures were designed and conducted according to the Guide for the Care and Use of Laboratory Animals published by National Institutes of Health (NIH Publication Number 80-23, revised 1996).

### 2.3. EAP Intervention

Prior to the MIRI experiment, rats among ES, NS, EA, and NA groups were pretreated with electroacupuncture for a total of 12 days in the waking state, with one day of rest after six consecutive days of treatment. Both EA and ES groups were pretreated at the PC6, located in the forelimbs [25]. Two acupuncture needles were separately inserted into the PC6 located on each upper limb which were located at a point 1.5 cm proximal to the palm crease just above the median nerve as previously described [16, 25], and an electrical current was provided to the needles for a period of 20 minutes through an electrical stimulator with a stimulus isolation unit (Han Acuten, WQ1002F, Beijing, China) at 2/15 Hz, with an intensity level of 1 mA [20]. The acupuncture needle, 7 mm long and 0.16 mm in diameter, was inserted 2-3 mm into the subcutis. In the NA and NS groups, the same pretreatments were applied at nonacupoint, located at the junction between the tail and buttock [27, 29]. Rats in SO and I/R groups were restrained in tubes the same as others groups for 20 min daily for 12 days. The electroacupuncture procedure was carried out by a specialized acupuncturist.

### 2.4. In Vivo MIRI

Rats that had accepted 12 sessions of EAP were anesthetized with mechanical ventilation using 5% isoflurane and maintained by inhalation of 1-2% isoflurane in 100% oxygen with a mixture of 70% N_2_O and 30% O_2_ after endotracheal intubation. Tidal volume and respiratory rate were set at 1.0 mL/100 g body weight and 45–60 breaths per minute, respectively. Adequate anesthesia was ensured by monitoring heart rate and the absence of a withdrawal response to a paw pinch. Lead II electrocardiogram (ECG) was monitored and recorded. The left carotid artery was cannulated for blood pressure measurement. Body temperature was maintained with a servo-controlled heating pad at 37°C.

The heart was exposed via a left thoracotomy between the 4th and 5th intercostal space. Following pericardiotomy, the left anterior descending (LAD) coronary artery was ligated with a 6.0 silk suture for 30 min to induce ischemia, followed by 240 min of reperfusion [29]. Successful coronary artery occlusion was confirmed by elevation of the ST segment in the ECG and an immediate 15–30 mmHg fall in arterial blood pressure. The chest was closed 30 min after the LAD was reperfused, and the rats were kept warm and allowed to recover. Buprenorphine HCl (0.05 mg/kg) was employed to minimize pain and distress by intramuscular injection after operation immediately [30].

### 2.5. Electrocardiogram (ECG) Recording and Scoring of Arrhythmia

A standard limb lead II ECG was successively monitored and recorded before, during myocardial ischemia, and after reperfusion under anesthesia by the use of a computerized PowerLab system (ADInstruments, Australia), and then monitored again from 210 min to 240 min under anesthesia [27]. The arrhythmias were assessed during the 30 min period of ischemia and the 30 min of reperfusion. The arrhythmia scores were accessed by an electrocardiogram specialist physician and assigned as described previously [27] according to the system described by Curtis and Walker [31] as follows: 0 = no arrhythmia; 1 ⩽ 10 s premature ventricular contraction (PVC) and/or ventricular tachycardia (VT); 2 = 11–30 s PVC and/or VT; 3 = 31–90 s PVC and/or VT; 4 = 91–180 s PVC and/or VT or reversible ventricular fibrillation (VF) of <10 s; 5 ⩾ 180 s PVC and/or VT, >10 s reversible VF; and 6 = irreversible VF. In addition, the survival rate of animals was also calculated.

### 2.6. Myocardial Enzyme Analysis

Following a four-hour reperfusion period, blood was collected from the jugular vein and centrifuged (10,000 g, 10 minutes, and 4°C). Serum lactate dehydrogenase (LDH), creatine kinase (CK) and creatine kinase-MB (CK-MB) levels were analyzed with a biochemistry analyzer. Plasma was extracted and analyzed for cTnT levels by electrochemiluminescence immune-assay method. All the myocardial enzyme detections were performed by the Medical laboratory of Jiangsu Province Hospital (Nanjing, China).

### 2.7. Histological and Morphological Analyses

After sacrificing the animals through intravenous injection of high-dose (150 mg/kg) of pentobarbitone at the end of the experiment, cardiac tissues were fixed in 4% paraformaldehyde (Sigma-Aldrich, Inc.) and embedded in paraffin. The embedded tissues were sectioned and stained with TUNEL assay kit as described previously [32, 33], which was employed to observe the positive apoptotic cells in myocardial tissue.

### 2.8. Apoptotic Molecules Analysis

After sacrificing the rats, the hearts were quickly removed and placed into liquid nitrogen. Then, total proteins were extracted for the further western blot (WB) assays. Our study has shown that electroacupuncture at PC6 could change gene expression profiles of I/R heart, and that EAP can markedly modify the expression levels of numerous genes involved in apoptotic pathways [27]. Therefore, in this study, we examined the protein expression of caspase 3, cytochrome c (Cyt c), Smac/Diablo, HtrA2/Omi, Endo G, AIF, Bcl2, BclxL, and Xiap to evaluate the status of apoptotic signaling.

### 2.9. Western Blot Analysis

WB was performed as described previously [34]. Briefly, after detecting the concentrations by the BCA protein assay (Pierce), equivalent amounts of protein (30 *μ*g/lane) were separated by SDS-polyacrylamide gel electrophoresis and transferred onto PVDF membranes. Each PVDF membranes were incubated with the appropriate primary antibodies anti-Cyt c (1 : 1000), anti-Smac/Diablo (1 : 1000), anti-HtrA2/Omi (1 : 1000), anti-Endo G (1 : 1000), anti-AIF (1 : 1000), anti-caspase 3 (1 : 1000), anti-cleaved caspase 3 (1 : 1000), anti-Xiap (1 : 1000), anti-BclxL (1 : 1000), anti-Bcl2 (1 : 1000), and anti-GAPDH (1 : 2000) and followed by incubation with peroxidase-conjugated secondary antibodies. Proteins were quantified using the SuperSignal West Pico Chemiluminescent substrate (Pierce).

### 2.10. Statistical Analysis

All values were expressed as a mean ± standard deviation (SD). Statistical analyses were performed using SPSS 17.0. Multiple group comparisons were performed by one-way ANOVA. Tukey's procedure was used for multiple-range tests. Differences were considered significant when *P* < 0.05.

## 3. Results

### 3.1. EAP at PC 6 Protected Rat Myocardium from MIRI

**Figure 2:**
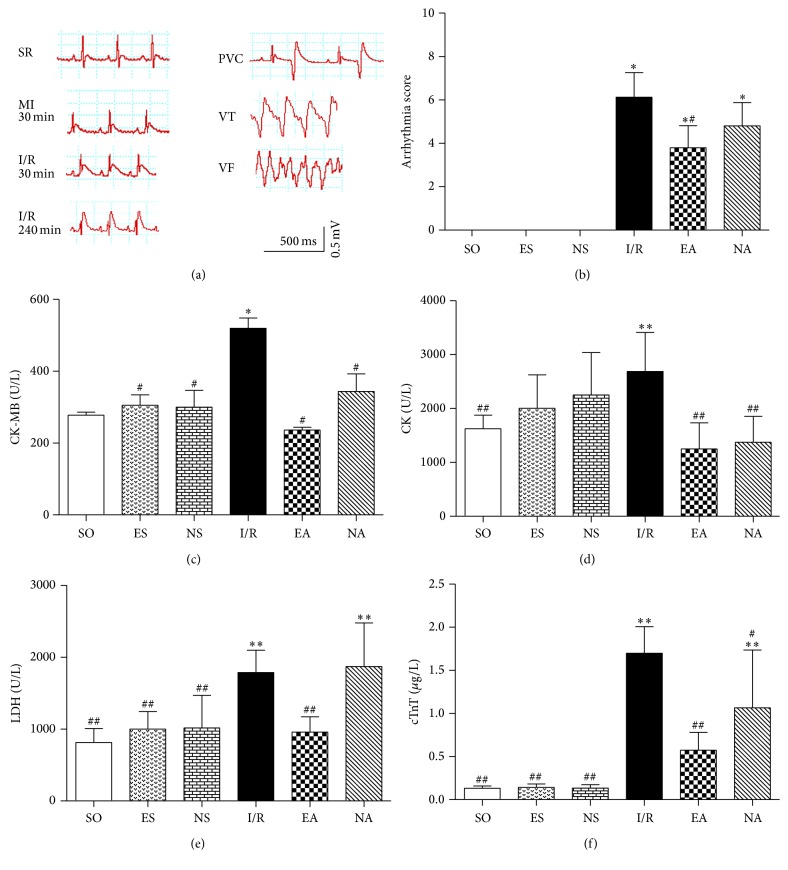
Effects of EAP on MIRI. (a) Representative ECG recording in standard limb lead II. PVC, premature ventricular contraction, VT, ventricular tachycardia, and VF, ventricular fibrillation. (b) Quantitative analysis for arrhythmia sores in each group. (c–f) CK, CK-Mb, LDH, and cTnT levels by biochemistry analyzer after myocardial I/R experiment in each group. Data were expressed as mean ± SD, *n* = 8–15/each group. ^*∗*^ *P* < 0.05 and ^*∗∗*^ *P* < 0.01 versus SO group; ^#^ *P* < 0.05 and ^##^ *P* < 0.01 versus I/R group.

To investigate protective effects of EAP at the PC6 on MIRI, we first observed ECG's ST segments, arrhythmic scores, and serum enzyme levels. The ECG recordings showed that the ST segments were noticeably elevated in I/R, EA, and NA groups during the 30 min after myocardial ischemia (MI) operation, and premature ventricular contraction (PVC), ventricular tachycardia (VT), and ventricular fibrillation (VF) were observed during the four-hour reperfusion period (Figure 2(a)), suggesting a successful I/R model. The arrhythmia scores were decreased in both EA and NA groups, especially in EA group compared to that in the I/R group (Figure 2(b)). The (LDH, levels of serum enzymes CK, CK-Mb, and cTnT), which reflect acute myocardial damage, significantly increased in the I/R group compared to the SO group, but returned to the levels of the SO group after EAP (*P* < 0.05) (Figures 2(c)–2(f)). Meanwhile, the survival rate in the EA group increased significantly to above 70%, compared to 50% in the I/R group (Figure S1 in Supplementary Material available online at http://dx.doi.org/10.1155/2016/4609784).

### 3.2. EAP at PC6 Promoted Survival of Cardiomyocytes against I/R Injury

**Figure 3:**
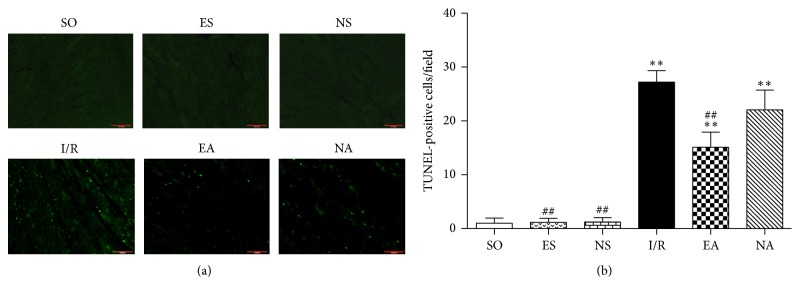
EAP promoted cardiomyocyte survival. (a) Representative images of TUNEL staining of each group. Green dots indicate TUNEL-positive cells. (b) Quantification of TUNEL-positive cells in each group. Data were expressed as mean ± SD, *n* = 8–15/each group. ^*∗∗*^ *P* < 0.01 versus SO group; ^##^ *P* < 0.01 versus I/R group.

Cardiomyocyte survival is a vital factor for cardiac function. In the face of I/R injury, the myocardium has the ability to develop numerous strategies to evade apoptosis. Expectedly, our study detected noticeably increased TUNEL-positive cells in the I/R heart, but the number of these cells was significantly decreased in the EA group (Figures 3(a) and 3(b)). Meanwhile, we also assessed the extent of myocardial fiber damage by ferroalumen hematoxylin staining [35, 36]. Our results showed that injury of myocardial fibers was obvious in I/R group, in which the cells were stained black. Myocardial cells from the EA group, however, did not pick up the stain (Figure S2).

### 3.3. EAP at PC6 Promotes Antiapoptotic Signaling and Inhibits Proapoptotic Signaling

**Figure 4:**
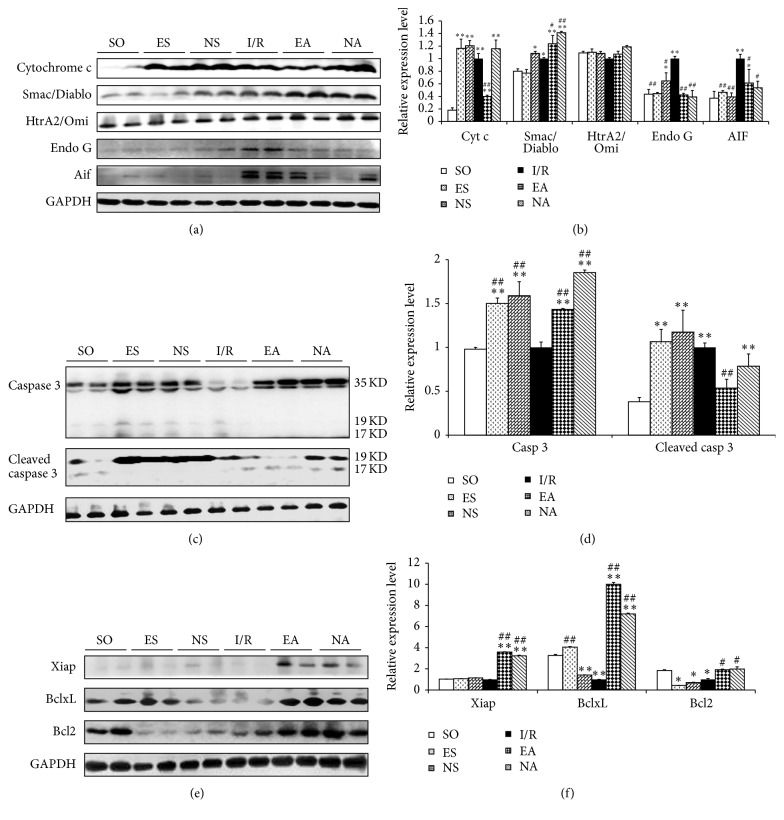
EAP regulated apoptotic signaling. Representative western blot results of proapoptotic, antiapoptotic, and histone acetylation proteins in each group ((a), (c), and (e)). Quantitative analysis of proapoptotic, antiapoptotic, and histone acetylation proteins in each group ((b), (d), and (f)). Data were expressed as mean ± SD, *n* = 8–15/each group. ^*∗*^ *P* < 0.05 and ^*∗∗*^ *P* < 0.01 versus SO group; ^#^ *P* < 0.05 and ^##^ *P* < 0.01 versus I/R group.

Apoptosis plays an important role in lethal reperfusion injury as indicated by numerous studies in various animal models [6, 37]. To further verify what we observed in the previous study by RNA-seq that apoptosis is a possible mechanism relevant to the myocardioprotective effects of EAP, caspase-dependent and caspase-independent apoptotic signaling in the heart tissues was examined by western blotting for caspase 3, Cyt c, Smac/Diablo, HtrA2/Omi, Endo G, and AIF. As shown in Figures 4(a) and 4(b), the expression of proteins of Cyt c, Endo G, and AIF increased in the I/R group but markedly decreased in the EA group (*P* < 0.05), suggesting an antiapoptotic mechanism involved in the cardioprotective effects of EAP against I/R injury. Interestingly, though the expression level of total caspases 3 was decreased in the I/R heart and EAP increased it significantly, the level of cleaved caspase 3, a biologically active caspase 3 enzyme, was elevated in the I/R group, and was evidently decreased in the EA group (*P* < 0.05, Figures 4(c) and 4(d)). Similarly, we detected reduction in antiapoptotic molecules, including Xiap, BclxL, and Bcl2 upon I/R injury, and a significant increase in these proteins after EAP (*P* < 0.05, Figures 4(e) and 4(f)).

## 4. Discussion

Acupuncture at PC6 is generally prescribed for the treatment of heart and chest disease symptoms, such as palpitation, chest distress, thoracalgia, gastralgia, and nausea and vomiting, based on the theory of Chinese medicine. Previous research has indicated that acupuncture at PC6 acupoint can attenuate cardiac injury, through reducing arrhythmias, apoptosis, and myocardial enzymes [16, 18, 20, 38, 39]. Our previous studies had demonstrated that electroacupuncture could effectively promote angiogenesis and protect myocardial tissue against ischemia injury [33]. EAP, one of acupuncture treatment approaches, has been demonstrated to be protective against I/R injury in brain or heart of the animal models [16, 25, 26, 40, 41]. The cardioprotective effects of EAP in patients undergoing heart valve replacement surgery or PCI operation have also been proved in a randomized controlled trial [22, 28], but the mechanism remains unclear.

As we all know, myocardial injury is often accompanied by changes in ECG and serum enzymes. ST segment elevation and heart rate disorders appear to be the most typical in the ECG detection. And the levels of serum enzymes (LDH, CK, and CK-Mb), especially cTnT [42], reflect acute myocardial damage effectively [43]. Our study, by using rat I/R model, suggested that EAP increased survival rate, reduced the ST segment, the arrhythmia score, and the release of serum enzyme from myocardium, alleviated myocardial fiber damage, and reduced the infarct size of MIRI measurement by TTC staining (the corresponding period results have published) [27], therefore protecting myocardial cells from I/R injury. These findings were consistent with the previous studies [16, 25, 37].

Our previous study and the literature have demonstrated numerous mechanisms that may contribute to MIRI [5, 27, 41–46], such as necrosis, apoptosis, and dysfunction of organelles. Apoptosis has been known to play an important role in the initial stages of reperfusion injury and serve as a mechanism of cellular self-destruction for a variety of processes [36]. Our data indicated a marked decrease in the number of TUNEL-positive cells as a result of EAP, and the extent of myocardial fiber damage was relieved in the EA group compared with the I/R group (Figure S2). The results suggested that EAP can effectively protect the myocardial cells from death, consistent with our previous studies on the reduction of myocardial infarction area by EAP [27]. Apoptosis is initiated by unbalancing pro- and antiapoptotic machineries [47–49]. Many studies have demonstrated the importance of caspase-dependent cell death pathways in injuries or diseases [50]. The most widely studied form of intrinsic apoptosis is stress-mediated release of Cyt c from the mitochondria that results in the formation of the apoptosome, which leads to the activation of the executioner caspase 3 [50]. The process may be regulated by Smac/Diablo and HtrA2/Omi. Meanwhile, it has also been discovered that in response to apoptotic stimuli, mitochondria can release caspase-independent cell death effectors such as apoptosis inducing factor (AIF) and Endonuclease G (Endo G) [51], resulting in caspase-independent apoptosis. In the caspase-dependent pathway, cleaved caspase 3-mediated signaling, such as Cyt c, Smac/Diablo, and HtrA2/Omi, directly activates cell death, whereas caspase-independent pathway, mainly including Endo G and AIF, causes cell death through DNA cleavage [52]. Our results showed that EAP decreased the Cyt c level and indirectly inhibited caspase 3 activation. EAP can also promote the expression of Smac/Diablo but not HtrA2/Omi, which indirectly negatively regulates caspase 3 via the inhibitors of apoptosis proteins (IAPs) [50, 53]. Additionally, this is, to the best of our knowledge, the first report of EAP exerting a protective role on myocardial I/R injury through inhibition of the expression of Endo G and AIF. As reported in other studies, the mitochondrial permeability transition pore (MPTP) is a central player of cell fate and is responsible for mitochondrial swelling with the consequent release of proapoptotic factors (i.e., Cyt c, AIF, and Smac/Diablo) [54]. When a large population of mitochondria is involved, these organelles are subjected to swelling, which leads to the release of the apoptotic factors [55]. In the present study, we demonstrated that EAP significantly decreased the opening of MPTP (Figure S3), therefore reducing the release of proapoptotic factors from the I/R heart. Unexpectedly, increased proteins expression levels of some proapoptotic molecules (Cyt c and cleaved caspase 3) and decreased antiapoptotic proteins (Xiap and Bcl2) were observed in the ES and NS groups, at a similar pattern in the I/R group. This observation needs to be addressed mechanistically in our future study, but it suggests that needling at nonacupoint might be also stimulation for the body to respond to certain extent, though the effectiveness of NA was not significant. This result is consistent with our previous finding [27].

The death of a cell was associated with autophagy and MAPK signaling. Early studies have indicated that apoptosis is caused by the activation of an autophagic process, which could be inhibited by chemical inhibitors of autophagy, and it was shown to depend on the autophagic genes APG5 and beclin [56]. We detected decreased beclin1 protein level in the heart treated by EAP (Figure S4). We also observed activated signal transduction of mitogen-activated protein kinases (MAPKs) in myocardial tissue, which had been reported as an important mediator of ischemic-related events [57]. The levels of p-JNK, p-P38, p-P44/42, and Ras were upregulated, and p-P38 level was downregulated in the I/R group, and all of them were reversed by undergoing EAP (Figure S5). Furthermore, to a certain extent, the EAP at nonacupoint also results in similar changes at molecular level as seen in the EA group (Figure 4), but its cardiprotective effect was inferior to the EA group. This is consistent with our previous observation [27], in which we described that EAP at nonacupoint modified less functional pathways, though it regulated some gene expressions to certain extent. The detailed mechanisms of different gene and protein expression patterns resulting from pretreatment on myocardial I/R injury using acupuncture at acupoint and nonacupoint remain to be investigated.

Interestingly, EAP on the sham operation rats resulted in increased expression of cleaved caspase 3, Cyt c, and Ras. However, these changes were not correlated with any pathological phenotype even though the levels of these proteins were as high as those in the I/R rats. This finding also is consistent with our previous study on ischemia-induced myocardial injury [27]. This phenomenon suggested that EAP might produce a similar stimulation on the heart, as does myocardial injury, mimicking an ischemic preconditioning or remote conditioning [44, 58–60]. A further study might be of significance to confirm this observation and explore its mechanism. Meanwhile, the experimental model is affected by a phenomenon called “nonreflow,” which can influence the results [61]. The nonreflow is a condition where there is a collapse of the blood vessel even after coronary artery opening, and it can limit the results. Therefore, it is necessary to pay more attention to the nonreflow in the follow-up study.

## 5. Conclusions

In summary, the present study demonstrates that EAP effectively protects the myocardium from I/R injury. And suppression of proapoptosis and promotion of antiapoptosis contribute to this effect. Thus, EAP could be an appropriate alternative treatment for patients with MI who will receive thrombolytic therapy or primary percutaneous coronary intervention.

## Supplementary Material

To investigate protective effects of EAP on MIRI, we evaluated rat survival rate and myocardial fiber injury. Our results showed that in the EA group, the survival rate was increased significantly (Fig S1), and the extent of myocardial fiber damage was relieved compared with the I/R group (Fig S2). Meanwhile, to assess the relationship between pro-apoptotic factors and the mitochondrial permeability transition pore (MPTP), we observed that EAP significantly decreased the opening of MPTP (Fig S3), therefore reduced the release of pro-apoptotic factors from the IR heart. In additional, we detected decreased beclin1 protein expression in the heart (Fig S4) and increased activity of signal transduction of mitogen-activated protein kinases (MAPKs) (Fig S5) after EA pre-treatment.
Figure S1 The survival rate of rats in each group (n=15). The survival number of each group was recorded after operation, and the survival rate of each group was calculated with the formula: (survival rat number/the total rat number) ×100%.
Figure S2 EAP at PC6 protected myocardial fibers against I/R injury. These images represent ferroalumen hematoxylin staining for myocardial fibers in each group.
Figure S3 EAP at PC6 decreased the opening of mitochondrial permeability transition pore. Mitochondrial PTP opening was assayed by fluorescence spectrophotometer, and data were expressed as means±SD, n=8-15 /each group. ∗, P<0.05vs. SO group; #, P<0.05 vs. I/R group.
Figure S4 EAP at PC6 decreased autophage-associated beclin1 expression level. A. Representative western blot results of beclin 1 proteins in each group. B. Quantitative analysis of beclin 1 protein in each group. Data were expressed as means±SD, n=8-15/ each group. ∗, P<0.05 vs. SO group; #, P<0.05 vs. I/R group.
Figure S5 EAP at PC6 influenced the expression levels of MAPK signaling. A. Representative western blot results of p-JNK, p-P38, p-P44/42, and Ras proteins in each group. B. Quantitative analysis of p-JNK, p-P38, p-P44/42, and Ras proteins in each group. Data were expressed as means ± SD, n=8-15/ each group. ∗, P<0.05 vs. SO group; #, P<0.05 vs. I/R group.
