# Supplementary material for: Cardioprotective Effect of Electroacupuncture Pretreatment on Myocardial Ischemia/Reperfusion Injury via Antiapoptotic Signaling
Source: Evid Based Complement Alternat Med. 2016 May 25;2016:4609784. doi: 10.1155/2016/4609784 (PMC4897718; doi:10.1155/2016/4609784)
Supplement: Supplementary file 1 — To investigate protective effects of EAP on MIRI, we evaluated rat survival rate and myocardial fiber injury. Our results showed that in the EA group, the survival rate was increased significantly (Fig S1), and the extent of myocardial fiber damage was relieved compared with the I/R group (Fig S2). Meanwhile, to assess the relationship between pro-apoptotic factors and the mitochondrial permeability transition pore (MPTP), we observed that EAP significantly decreased the opening of MPTP (Fig S3), therefore reduced the release of pro-apoptotic factors from the IR heart. In additional, we detected decreased beclin1 protein expression in the heart (Fig S4) and increased activity of signal transduction of mitogen-activated protein kinases (MAPKs) (Fig S5) after EA pre-treatment. Figure S1 The survival rate of rats in each group (n=15). The survival number of each group was recorded after operation, and the survival rate of each group was calculated with the formula: (survival rat number/the total rat number) ×100%. Figure S2 EAP at PC6 protected myocardial fibers against I/R injury. These images represent ferroalumen hematoxylin staining for myocardial fibers in each group. Figure S3 EAP at PC6 decreased the opening of mitochondrial permeability transition pore. Mitochondrial PTP opening was assayed by fluorescence spectrophotometer, and data were expressed as means±SD, n=8-15 /each group. ∗, P<0.05vs. SO group; #, P<0.05 vs. I/R group. Figure S4 EAP at PC6 decreased autophage-associated beclin1 expression level. A. Representative western blot results of beclin 1 proteins in each group. B. Quantitative analysis of beclin 1 protein in each group. Data were expressed as means±SD, n=8-15/ each group. ∗, P<0.05 vs. SO group; #, P<0.05 vs. I/R group. Figure S5 EAP at PC6 influenced the expression levels of MAPK signaling. A. Representative western blot results of p-JNK, p-P38, p-P44/42, and Ras proteins in each group. B. Quantitative analysis of p-JNK, p-P38, p-P44/42, and R [file 4609784.f1.doc]

**Supplementary material**

**
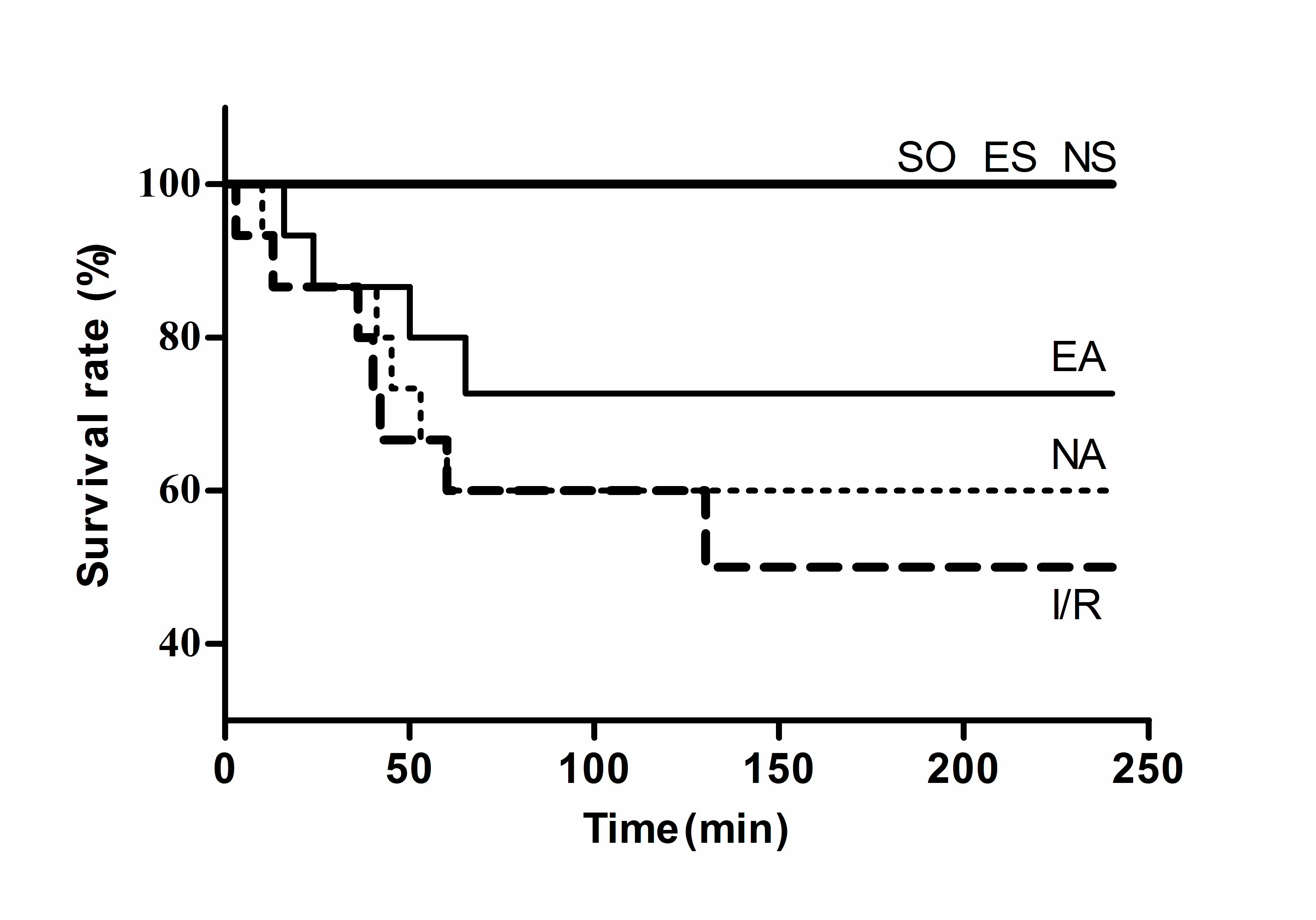
**

**Figure S1 The survival rate of rats in each group (n=15)**. The survival number of each group was recorded after operation, and the survival rate of each group was calculated with the formula: (survival rat number/the total rat number) ×100%.

**
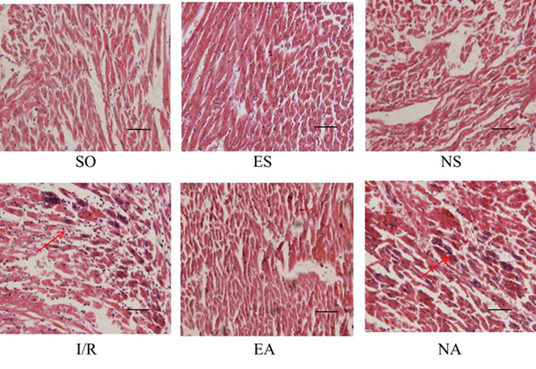
**

**Figure S2 EAP at PC6 protected myocardial fibers against I/R injury.** These images represent ferroalumen hematoxylin staining for myocardial fibers in each group.


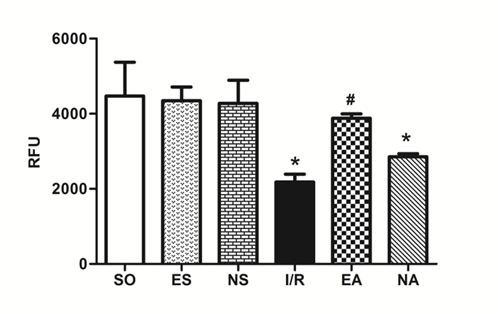


**Figure S3 EAP at PC6 decreased the opening of mitochondrial permeability transition pore.** Mitochondrial PTP opening was assayed by fluorescence spectrophotometer, and data were expressed as means±SD, n=8-15 /each group. *, P<0.05, **, P<0.01 vs. SO group; #, P<0.05, ##, P<0.01 vs. I/R group.

**A**


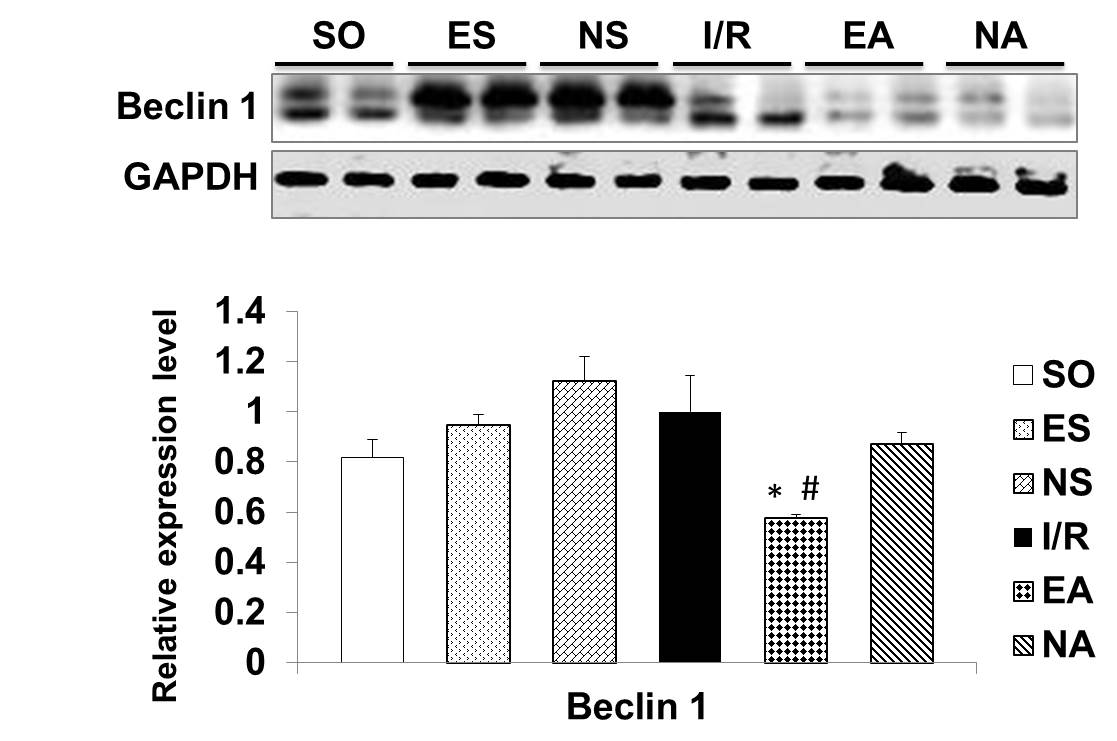


**B**

**Figure S4 EAP at PC6 decreased autophage-associated beclin1 expression level.**

A. Representative western blot results of beclin 1 proteins in each group. B. Quantitative analysis of beclin 1 protein in each group. Data were expressed as means±SD, n=8-15/ each group. *, P<0.05, **, P<0.01 vs. SO group; #, P<0.05, ##, P<0.01 vs. I/R group.


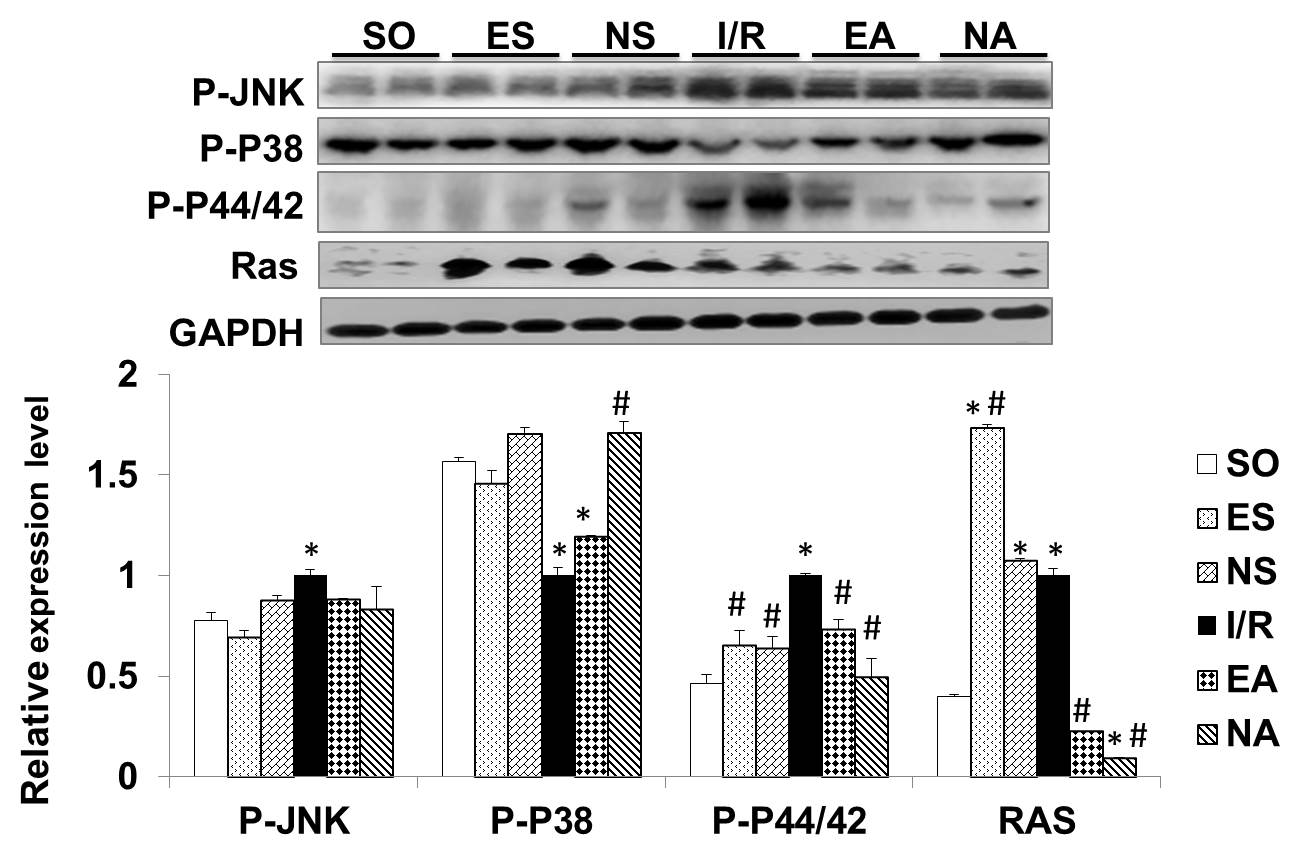


**A**

**B**

**Figure S5 EAP at PC6 influenced the expression levels of MAPK signaling.** A. Representative western blot results of p-JNK, p-P38, p-P44/42, and Ras proteins in each group. B. Quantitative analysis of p-JNK, p-P38, p-P44/42, and Ras proteins in each group. Data were expressed as means ± SD, n=8-15/ each group. *, P<0.05, **, P<0.01 vs. SO group; #, P<0.05, ##, P<0.01 vs. I/R group.
